# Orientation of internet use, government trust, and public environmental satisfaction an empirical study in Mainland China

**DOI:** 10.1371/journal.pone.0287340

**Published:** 2023-10-26

**Authors:** Jinghuan Chen, Li Yang, Lijuan Zheng

**Affiliations:** 1 Southwestern University of Finance and Economics, Chengdu, Sichuan, China; 2 Sichuan Jiangyou City Notary Public Office, Chengdu, Sichuan, China; Jiangsu University, CHINA

## Abstract

Public environmental satisfaction is related to the healthy living standard of human beings and sustainable development of an economic society. In the context of the continuous updating of Internet technologies, it is necessary to study the correlation between Internet use and public environmental satisfaction, but few studies have focused on the effect of the orientation of Internet use on public environmental satisfaction, and its mediating mechanisms. This study considered survey data from the China Social Survey 2019 which were conducted in a sample of 5,112 residents, SPSS 16.0 and ordinary least squares regression model was used to analyze the relationship between orientation of Internet use, government trust and public environmental satisfaction. The results showed politically-oriented Internet use was positively correlated with public environmental satisfaction, and entertainment-oriented Internet use did not correlate with public environmental satisfaction. Furthermore, government trust partially mediated the relation between politically-oriented Internet use and public environmental satisfaction. Several implication policies are suggested for improving public environmental satisfaction. This study takes into account individual initiative and government’s control of information during Internet use, emphasizes the impact of different individual Internet use preferences on environmental satisfaction in the web environment with strong government control. By incorporating individual factors at the micro level and social factors at the macro level, this paper is in order to improve public environmental satisfaction.

## 1. Introduction

With the development of economic level, ecological and environmental problems are becoming more and more prominent, and China is no exception, also facing serious environmental problems. Haze in winter, dust storms in spring, and the ever-present water pollution pose serious threats to the life and health of residents. Along with the improvement of network information technology, these pollution incidents have been spread and fermented by the Internet and other media, which has sparked the attention of the public and the government.

A good ecological environment plays an important role in enhancing people’s sense of well-being and security in life, which is both an objective fact and a perception. Based on the perspective of perception, public environmental satisfaction becomes an important expression of ecological environment status. Public environmental satisfaction is the public’s subjective evaluation of environment, i.e., the extent to which the public believes that the state of the environment meets its expectations or needs [1]. Although by definition, public environmental satisfaction is a subjective content, influenced by the subjective judgment of individuals, individuals are part of society, and their views and values are influenced by multiple factors in society from a sociological point of view, a very important factor of which is the information they receive. Individuals get different information about the environment from Internet, it prompts them to form different perceptions and feelings. With the development of the Internet, news about ecological conditions, weather crises, environmental assessments, etc. spread on the Internet, the use of the Internet becomes an important dimension for individuals to obtain information and influence public environment satisfaction.

Previous studies have reported conflicting findings on the relation between Internet use and public environmental satisfaction. A part of the literature suggests that higher frequency of Internet use results in lower environmental satisfaction among the public [2]. Owing to its pluralistic and autonomous characteristics [3], new media such as the Internet may result in the large-scale dissemination of negative environmental information, thereby amplifying people’s dissatisfaction with the current environment [4]. Porumbescu surveyed 1100 Seoul citizens and revealed that Internet use reinforces the negative relation between public expectations of public sector performance and satisfaction with public services. Thus, Internet use significantly reduces public trust in government [5]. Some scholars have observed that the Internet positively affects environmental satisfaction among the public. For example, Deyan Peng and Yacheng Li asserted that the government promotes “environmental protection for the people” and that the public can learn about the government’s environmental protection work through the Internet, thereby helping people develop positive environmental attitudes [6]. Hong H. revealed that Internet use promotes a more cooperative relation between Internet users and the government. Internet users have more opportunities to participate in the government’s environmental protection work, which, in turn, enhances positive environmental attitudes, participation, and satisfaction among public [7].

The existing literature is controversial because of ignoring individual initiative and lacks research on types of Internet use. Most studies consider the individual as a passive endpoint on the Internet’s axis of influence. In fact, the Internet creates an “echo chamber”, in which individuals who use the Internet selectively view information that matches their interests and overlook information that they disagree with, reinforcing their original views [8]. Therefore, individual initiative should also be considered, that is, the specific role of the Internet should be examined from the perspective of individual Internet use. The purpose of this study is distinguishing the orientation of Internet use and exploring the direct and indirect relationships between different orientations of Internet use and public environmental satisfaction.

Public environmental satisfaction is not only a reflection of the ecological environment, but also an evaluation of the government ability and government behavior, especially in heavily polluted areas where the government behavior has a direct impact on the local ecological environment. From the perspective of perception, the concept of government trust is involved when the public believes that the government can pay attention to environmental issues and even sacrifice the interests of local economic development to support the development of environmental protection directly. Government trust could be understood as a state of positive expectation that the public can trust the government and its actions [9]. Government trust can influence public environmental satisfaction to some extent. For the government, a high level of public trust in the government will force the government to maintain its public image of environmental protection, further implement environmental governance practices, and improve environmental satisfaction [10]. Failure to take proactive measures to address environmental pollution will reduce government trust, weaken government legitimacy, and increase public discontent [11]. Government trust affects public satisfaction while also being influenced by multiple factors, among which mass media, including the Internet, play an important role. In the Internet society, information itself is more complex because of its pluralism, and the groups using the Internet are actively or passively involved in the production and creation of social demands, and in this process, their own perceptions and attitudes are influenced [12]. Government is the subject of publicity and supervision of Internet information and one of the subjects of Internet information content, the content and evaluation of Internet information expression affects the image of the government and the public’s trust in the government [13]. Based on this, this paper introduces government trust and analyzes whether it has a mechanism effect in the path of orientation of Internet use affecting public environmental satisfaction.

Through the above review of the practice context and literature, this paper focuses on two questions:

1. Whether different orientations of Internet use affect public environmental satisfaction consistent?
2. Is there a mechanism effect of government trust in the path of Internet use affecting environmental satisfaction?

With the escalation of information-based society, individuals’ Internet behavior has become more common in society. Therefore, along with active environmental governance, there is a need to analyze the impact and influence mechanisms of Internet use preferences, which is still a considerable research gap in this field. This study attempts to contribute to the literature and practice of public environmental satisfaction by examining the role of orientation of internet and the mediating and moderating role of government trust. This study provides insights into how the orientation of Internet use affect public environmental satisfaction, which is critical to understanding the effectiveness of government environmental governance in the Internet era. Thus, this study contributes to achieving increased public environmental satisfaction and improved environmental quality.

## 2. Literature review and hypothesis development

### 2.1 Media use and public environmental satisfaction

The relation between media use and public environmental satisfaction has been examined from two perspectives: the media virtuous circle theory and the media inhibition theory. The media virtuous circle theory holds that the media is a vital intermediary for the public to participate in social interactions and that it enhances the public’s intrinsic sense of efficacy, thereby developing the public civic consciousness and directly or indirectly increasing public satisfaction with government affairs [14]. The media inhibition theory holds that the negative news about the environment increases dissatisfaction and inhibits the positive effects of environmental management efforts [15].

On this basis, two types of empirical conclusions were developed. (1) Media use positively affects public environmental satisfaction. Kara Chan presented an optimistic and positive view of the role of the media, he mentioned that mass media improves people’s environmental behavior and green consciousness, promotes greater supervision of the public environment, compels government subjects to make efforts to improve the environment, and thereby enhances public environmental satisfaction [16]. (2) Media use negatively affects public environmental satisfaction [17]. Coleman opined that after being exposed to environment-related information disseminated by the media, the public becomes more acutely aware of the threat of environmental pollution and damage to personal life and health, which decreases environmental satisfaction [18]. In a study examining the quality of media coverage, Mazur concluded that even if the media objectively and neutrally reported on real environmental problems without emphasizing the potential risks, the widespread public attention to such reports significantly increases the public’s perception of environmental risks and decreases individuals’ environmental satisfaction [19].

The effects of media use on environmental satisfaction remain controversial, and the reason for the two dissimilar findings is the distinction between communication media considered by researchers and the content of information preferred by the public, leading to varied cognitive compositions [20]. Therefore, public media use and preferences should be refined.

### 2.2 Orientation of internet use and public environmental satisfaction

Communication scholars have classified different types of Internet use according to different criteria. Users are classified into four types according to the content of Internet use: (1) researchers, who use the Internet to send emails and conduct surveys; (2) consumers, who use the Internet for shopping and consumption; (3) expressers, who use the Internet to express their ideas and opinions; (4) entertainers, who use the Internet for entertainment and leisure activities such as games and music [21]. Based on internet usage process, collective intervention and individual intervention are two types. Under collective intervention, users share ideas and concepts with others through the Internet. Individual intervention is the act of immersive Internet use by the user alone [22]. In terms of information consumption, Internet use behavior is divided into two types: politically-oriented and entertainment-oriented. Politically-oriented behavior refers to users’ preference to use the Internet to learn about current affairs and public issues. By contrast, entertainment-oriented behavior refers to users’ preference to use the Internet for leisure and entertainment [23]. Clearly, the existing classifications discuss the preferences and purposes of individuals in Internet use. Researchers and expressers, as well as collective interventions, focus on politically-oriented Internet use. Consumers, entertainers, and individual interventions focus on entertainment-oriented Internet use. Given that the politically-oriented and entertainment-oriented classifications are more representative and complete, and Presents the user’s usage preference, this classification seems to be appropriate understand the types of Internet use in this study.

Studies focusing on the effects of politically-oriented and entertainment-oriented Internet use on environmental satisfaction are limited, some existing theories provide inspiration for further consideration. “Doctrine of empowerment” refers to a process, intervention, and practice that empowers individuals or groups and stimulates individual or group potential [24]. According to the doctrine of empowerment, the development of the Internet provides opportunities for empowerment, the empowerment of individuals or groups strengthens individual action and participation [25]. Scholars have examined Internet-empowered political participation and have revealed that with the development of online technologies, the Internet has facilitated people to express, share, and discuss their evaluations and perceptions of government performance, thereby strengthening their attitudes and satisfaction evaluations of government [26]. Boulianne opined that the Internet provides more social power to citizens and promotes their participation in social affairs and understanding of public affairs [27]. This part of the scholars, starting from empowerment theory, found that the Internet empowers citizens and promotes their voice and participation, thus affirming the positive role of the Internet. The “time substitution hypothesis” proposes that excessive use of media such as television and the Internet encroaches on people’s time to focus on public affairs and discourages them from expressing their views and making evaluations of public affairs and public goods. Putnam argued that excessive time spent watching television leads to a general indifference of the public to public affairs such as environment, health care, and hygiene [28]. Putnam further observed that with the popularity of the Internet, public more likely engaged in activities alone without communicating with others and expressing their views on matters in the public sphere [29]. In the opinion of this group of scholars, the time spent on media such as the Internet makes the public spend less time on public affairs.

The Chinese government tries to control the dissemination of news and information on the Internet to maintain social stability [30]. Some scholars have argued that unlike the “fourth power” in Western countries [31], the Chinese media is an “ideological state apparatus” through which the government shapes and directs public opinion by regulating the media [32]. In accordance with the gatekeeper theory, gatekeepers in the communication process only allow information that conforms with the mainstream values, group norms, and gatekeeper value standards to enter the communication channel [33]. White mentioned that there are various news events in society, the media does not and cannot report them all but selectively collects and reports the news through gatekeepers [34]. News content is jointly generated by media organizations and gatekeepers [35]. With regard to environment-related issues, officials prefer using mainstream media networks to spread a beautiful and harmonious ecological landscape. Furthermore, the public is restricted from communicating and discussing negative information on a large scale through the Internet [36]. From the empowerment doctrine, the Internet empowers individuals with the right to know and participate, thus enhancing public attention to public events, and combining with gatekeeper theory, Chinese Internet users are more likely to receive positive information about the environment and have higher environmental satisfaction during Internet use. According to the "time substitution hypothesis," the weaker attention to public affairs by online entertainment makes it difficult for some Chinese Internet users to receive positive information about the ecological environment, and the environmental satisfaction of this group is likely to decrease. Considering the above findings, the following hypothesis is proposed:

**Hypothesis 1 (H1):** *A positive and significant relation exists between politically-oriented Internet use and public environmental satisfaction*.

**Hypothesis 2 (H2):** *A negative and significant relation exists between entertainment-oriented Internet use and public environmental satisfaction*.

### 2.3 Mechanisms between internet use orientation and public environmental satisfaction

#### 2.3.1 Government trust and public environmental satisfaction

How does Internet use affect public environmental satisfaction? Trust is a powerful mediating determinant that needs further investigation [37]. The government plays a leading role [38], the level of trust in government, then, is crucial. Government trust is defined as citizens belief and confidence that the government or political system operates to produce results consistent with their expectations [39]. According to Miller, government trust should be understood as the public’s evaluation of public services or government performance [40]. Within this analytical framework, trust in government follows an “expectation-response” path: this implies that people expect government to meet their intended needs. Thus, the relation between trust in government and satisfaction with environmental is based on government performance and public expectations [41]. The government responds to public expectations by further implementing environmental governance practices and improving the quality of environmental public service products [10]. Chanley demonstrated that the public’s evaluation of the government’s environmental work is closely related to their trust in the government [42]. Examining empirical research data in Shanghai, Yi Chengzhi concluded that the public’s trust in the government enables the government to work toward improving public satisfaction with environmental public services [43]. Li Wenbin and Lai Linhui documented that public trust in government significantly and positively affects public satisfaction with the environment [44]. These studies have emphasized connotations such as satisfaction with government’s environmental governance work [45], satisfaction with habitat environment [46], and satisfaction with objective environmental quality [47]. Notably, government behavior affects the latter two types of satisfaction. Therefore, whether government trust positively affects public satisfaction should be verified.

#### 2.3.2 Orientations in internet use and government trust

The effect of Internet use on government trust is considered to be bi-directional: the negative effect is based on the “media inhibition theory,” that is, the focus on negative information about government departments and political tasks owing to the profit-driven commercial competition among the media deepens public dissatisfaction and apathy toward the government [48]. In Putnam’s view, media use compresses and crowds out public participation in public life, causing a decline in social capital and ultimately undermining public trust in government [49]. Su Zhenhua and Huang Waibin observed that the higher the frequency of Internet use, the lower the level of public trust in government [50]. The positive effect is based on the “media virtuous circle theory,” in which media use increases citizen accountability, political knowledge, political interest, and political involvement, thereby promoting political trust [51]. Ramona and Kathleen used Pew Internet and American Life Project survey data to analyze citizen-initiated contact with government. The authors suggested that the Internet can increase the frequency of citizen-government interactions and, more broadly, trust in and awareness of government [52].

The Chinese media context differs from that of the West, and the relevance of Internet use to government trust should be further clarified in terms of the gatekeeper theory. As mentioned previously, gatekeepers in the media allow positive information about the government to appear before Internet users. Moreover, the system of “party-controlled media” in China maintains public support for the government and increases public trust in the government [53]. Related studies have suggested that the Internet promotes scientific and democratic rationalization of public policies and improves the government’s credibility [54]. The political trust of college students exposed to official media such as Newswire and People’s Daily is higher [55]. Thus, politically- oriented Internet use provides more positive news and builds stronger political trust. By contrast, entertainment-oriented Internet use leads Chinese citizens to focus less on positive news and acquire some negative news from the Internet for circulation and ratings [56], thereby affecting personal trust in the government. Accordingly, the following hypotheses are proposed:

**Hypothesis 3:** *Politically-oriented Internet use is positively associated with public environmental satisfaction by increasing individuals’ trust in government*.

**Hypothesis 4:** *Entertainment-oriented Internet use is negatively associated with public environmental satisfaction by decreasing individuals’ trust in government*.

## 3. Materials and methods

### 3.1 Data sources

This study uses the data from the Chinese Social Survey in 2019 (CSS2019). The CSS2019 employed a multi-stage stratified probability sampling method, that is, sampling by counties/cities/districts, neighborhood committees/village committees, households, and residents. The survey area covered 31 provinces/autonomous regions/municipalities directly under the central government, including 151 counties (districts) and 604 residents (villages) committees. The questionnaire survey was conducted through household visits. The total number of households surveyed exceeded 10,000, and the respondents comprised households aged between 18 and 69 years, indicating a good national representation. Upon cleaning up the outliers and missing values, the size of the final valid sample for this research was 5,112. Table 1 shows the descriptive statistics of the participants.

**Table 1:** Descriptive statistics of the participants.

Variable	*n*	% or Mean(SD)
**Gender**		
male	2189	42.82
Female	2923	57.18
**age**		
Range:18–69	5112	46.39(14.18)
**Education level**		
illiteracy	465	9.08
elementary education	1152	22.54
secondary education	2535	49.59
higher education	960	18.79
**Type of residence**		
urban	2909	56.91
Rural	2203	43.09
**Political identity**		
party member of CPC	536	10.47
non-party personage	4576	89.53
**Nationality**		
Han Chinese	4702	92.00
non-Han	410	8.00
**Marital status**		
married	4113	80.48
unmarried	999	19.52

### 3.2 Variable design

#### 3.2.1 Dependent variable

The dependent variable is public environmental satisfaction, that is, the degree of public recognition of the current ecological environment, reflecting the suitability of the ecological environment and human social development. Question d5b in the CSS2019 questionnaire emphasizes environmental satisfaction. Specifically, the question was “Please use 1–10 points to express your satisfaction with the environmental conditions of your current residence. 1 point means very dissatisfied, 10 points means very satisfied.” Accordingly, the respondents indicated their scores on the environmental status of their residence based on their feelings. The higher the score, the higher the public’s satisfaction with the environment. Statistical calculation revealed that the average score of environmental satisfaction of Chinese public in CCS2019 was 6.84. More than 85% of the public were relatively satisfied with the environmental conditions of their current residences, and their scores were ≥5. Furthermore, less than 15% of the residents indicated relatively low satisfaction with the environmental conditions of their residences. In addition, environmental satisfaction is considered as a dichotomous variable in the robustness test of this study. If the satisfaction score was 1–5, the assigned value was 0, indicating low environmental satisfaction; if the satisfaction score was 6–10, the assigned value was 1, indicating high environmental satisfaction.

#### 3.2.2 Independent variable

The independent variable is the Internet use. Two items in the CSS2019 questionnaire focused on residents’ Internet use: D4a and D4b1. The D4a question was as follows: “Now the Internet is quite popular. You can use your mobile phone and computer to surf the Internet. Do you usually surf the Internet (such as watching news on your computer or mobile phone using WeChat and other activities)?” The residents were required to choose answer 1 if they had ever used the Internet and choose answer 2 if they had never used the Internet. The D4b1 question focuses on the frequency of the respondents’ seven types of Internet use content, which are as follows: 1. Browsing current political information (such as watching party and government news); 2. Entertainment (such as playing online games/listening to music/watching videos/reading novels); 3. Chatting and making friends (such as WeChat and other dating activities); 4. Business or work; 5. Learning and education; 6. Online shopping/life services (such as online shopping, takeout, map navigation, and map positioning); and 7. Others. The use frequency of topic setting was almost every day, more than once a week, at least once a week, at least once a month, several times a year, and never. The scores for the options from almost every day to several times a year were 1, 2, 3, 4, and 5 in turn, and the score for the “never” option was 0.

In the process of operationalization of the core explanatory variables, options were indexed and assigned in reverse: “almost every day = 5 points,” “multiple times a week = 4 points,” “at least once a week = 3 points,” “at least once a month = 2 points,” “several times a year = 1 point,” and “never = 0 points.” For the sample with “No Internet access” in question D4a, the value assigned was 0, implying that the group never had access to the Internet. Drawing on the classification of Internet use methods in existing literature, Internet use was divided into two types based on the type of Internet information consumption: politically-oriented and entertainment-oriented. Politically- oriented Internet use refers to the users’ awareness concerning current political news, social news, commercial news, and education news. Entertainment-oriented Internet use refers to users’ preferences for leisure and entertainment activities. The topics selected to measure politically-oriented Internet use included the following: 1. Browsing current political information (such as watching party and government news); 4. Business or work; and 5. Learning and education, with their scores forming a continuous variable of [0, 15]. The higher the score, the higher the frequency of respondents’ politically oriented Internet use. The topics for measuring entertainment-oriented Internet use included the following: 2. Entertainment and leisure (such as playing online games/listening to music/watching videos/reading novels); 3. Chatting and making friends (such as WeChat and other dating activities); and 6. Online shopping/life services (such as online shopping, takeout, map navigation, and map positioning). Their scores were added to form a continuous variable with a score of [0, 15]. The higher the score, the higher the frequency of respondents’ entertainment-oriented Internet use.

#### 3.2.3 Mediator variable

In this study, government trust is the mediator variable. Erikson and other scholars opined that government trust is the public’s evaluation and satisfaction with government procedures and policies [37]. Erikson’s definition of political trust (that is, the public’s confidence in the government’s service to the people) was used to examine the public trust in the Chinese government. The survey on government trust in the CSS2019 questionnaire was divided into three levels of government departments from top to bottom: central, district and county, and township governments. The question posed was as follows: “Do you trust the following institutions?” The options were “total distrust,” “less trust,” “comparative trust,” “very trust,” and “hard to say.” In the process of operationalization, government trust was treated as a fixed-distance variable, and the score of choosing “hard to say” was the average value. The values assigned were as follows: total distrust = 1, less trust = 2, comparative trust = 3, and very trust = 4. Thereafter, the scores of three levels of government departments were added to form a continuous variable of [3, 12]. The higher the score, the higher the degree of trust in the government.

#### 3.2.4 Other control variables

Other variables affecting public environmental satisfaction were controlled for in this study to control the estimation error caused by missing variables as much as possible. The age variable considered the number of respondents’ answered ages. In terms of gender, male = 1, female = 0. In terms of the education level, illiteracy = 1; primary school = 2; junior high school, senior high school, technical secondary school, vocational and technical school = 3; college, undergraduate, postgraduate, and other higher education = 4. In terms of location of residence, urban residence = 1 and rural residence = 0. In terms of nature of the unit, work within the system = 1 and work outside the system = 0. In terms of political identity, Communist Party of China (CPC) member = 1 and non-CPC member = 0. In terms of nationality, Han = 1 and non-Han = 0. In terms of marital status, married = 1 and unmarried = 0. Investigated regional variables were assigned values as follows: east = 1, middle = 2, and west = 3. Family population was a continuous variable. In terms of income, the bilateral tail was shrunk by 1% of the total income variables of the respondents in the last year and then entered the model in the form of natural logarithms. In this study, 1 was first added to the total income and the logarithm was taken to avoid too many missing values after taking the logarithm. The control variables at the macro level included the harmless treatment rate of domestic waste, per capita emission of nitrogen oxides, grassland area, the total amount of wastewater discharge, and the amount of general industrial solid waste, which were treated as continuous variables.

### 3.3 Analytical methods

First, given that the dependent variable was a continuous variable with a score of [1,10], the independent variable was also continuous. The ordinary least squares (OLS) regression model was used verify the relation between Internet use and public environmental satisfaction.

The OLS model is as follows:

$$environmental\_satisfaction=a_{0}+\beta_{0}*Internet+\gamma_{0}*X+\mu_{0},$$ (1)

where environmental_satisfaction denotes the individual’s environmental satisfaction score, Internet denotes the individual’s Internet use frequency score, *X* denotes the control variable, and *μ*_0_ denotes the stochastic disturbance term.

Second, the intermediary mechanism through which Internet use affects public environmental satisfaction was analyzed. In this study, government trust was considered as a mediator variable, and the stepwise regression method was used to identify the mediating effect. The model of stepwise regression method is as follows:

$$environmental\_satisfaction=a_{0}+\beta_{0}*Internet+\gamma_{0}*X+\mu_{0}$$ (1)

$$Mechanism=a_{1}+\beta_{1}*Internet+\gamma_{1}*X+\mu_{1}$$ (2)

$$environmental\_satisfaction=\alpha_{0}^{\prime }+\beta_{1}^{\prime }*Internet+\beta_{2}*Mechanism+\gamma_{2}*X+\mu_{2}$$ (3)

The meaning of variables in Eq (1) is the same as above, and Eq (2) indicates the effect of Internet use on individuals’ trust in government; Mechanism denotes the mediating variable. In Eq (3), which indicates the mediating effect, the coefficient β_2_ denotes the effect of mediator on the dependent variable, and $\beta_{1}^{\prime }$ denotes the effect of Internet use on the independent variable under the premise of controlling the mediating effect. *X* and *μ* denote control variables and stochastic disturbance terms, respectively.

## 4. Results

### 4.1 Descriptive statistics

Table 2 presents the descriptive statistical results. In terms of the dependent variable, the average score of public environmental satisfaction was 6.84 in 2019 in China, and the standard deviation was 2.17. This result indicates that public environmental satisfaction is generally high in China. In terms of the independent variable, the average value of politically-oriented Internet use was 5.10 and the average value of entertainment-oriented Internet use was 6.24. This result indicates no obvious two-level difference in the use of Internet content by the public in China.

**Table 2:** Descriptive statistics (N = 5112).

Variable Name	Variable Definition	Mean Value		Standard Deviation	Minimum Value	Maximum Value
**The dependent variable**						
Environmental satisfaction	Continuous variables 1–10 points	6.84		2.17	1	10
**The independent variables**						
Politically-oriented Internet use	Continuous variables 0–15 points		5.10	5.16	0	15
Entertainment-oriented Internet use	Continuous variables 0–15 points	6.24		5.28	0	15
**Control Variables**						
Age	2019 minus year of birth	46.41		14.17	18	68
Gender	Male = 1; Female = 0	0.43		0.50	0	1
Type of residence	Urban = 1, rural = 0	0.57		0.49	0	1
Political identity	CPC member = 1, non CPC member = 0	0.11		0.31	0	1
Nationality	Han Chinese = 1, non-Han = 0	0.92		0.27	0	1
Marital status	Married = 1, unmarried = 0	0.80		0.39	0	1
Nature of the unit	Public-sector job = 1, non public-sector job = 0	0.09		0.26	0	1
Education level	Illiteracy = 1, primary education = 2, secondary education = 3, higher education = 4	2.78		0.85	1	4
Annual personal income	Continuous variable (logarithm)	8.63		3.68	0	12.6
Family population	Continuous variable	6.28		1.42	3	8
Region	East = 1, Middle = 2, West = 3	1.81		0.81	1	3
Harmless treatment rate of domestic waste	Continuous variable (%)	98.95		2.84	86.9	100
NOx emissions per capita	Continuous variable (ton)	0.009		0.003	0.005	0.02
Total wastewater discharge	Continuous variable (10,000 tons)	330626		216139	27115	882020
Output of industrial solid waste	Continuous variable (10,000 tons)	12813		9000	437	34162
Grassland area	Continuous variable (hectares)	7378		12481	73	78804
**Mediator**						
Government trust	Continuous variable	9.45		1.87	4	12

### 4.2 Benchmark regression analysis

Table 4 presents the benchmark regression analysis results. Models 1, 2, and 3 are the results of multiple linear regression analysis, and models 4, 5, and 6 are the results of binary logistic regression. The data in the table indicate that after controlling for the effect of individual characteristics and macro environmental factors, the effect of politically-oriented Internet use on public environmental satisfaction was significantly positive at the significance level of 1% in both OLS regression and logistic regression. However, the regression results of entertainment-oriented Internet use differ in the two regression models: in the OLS regression model, the entertainment-oriented Internet use mode did not significantly affect public environmental satisfaction and only had a positive correlation in a statistical sense. The binary logistic regression results revealed a positive correlation at 5% significance level. This statistical result reveals that the current politically oriented Internet use significantly positively affected environmental satisfaction of the public in China, that is, the higher the frequency of politically-oriented Internet use, the higher the environmental satisfaction of the public. Thus, H1 of this study is verified, the findings support the “doctrine of empowerment”. Notably, the effect of entertainment-oriented Internet use on environmental satisfaction was not significant in the multiple regression model, but a positive statistical significance existed. In the binary logistic regression model, a significant positive correlation was observed. The reasons for this difference in results should be further studied. The regression results of Model 3 indicate that when the politically-oriented Internet use and entertainment-oriented Internet use modes were included in the model and controlled, the politically-oriented Internet use mode significantly positively affected environmental satisfaction, but the entertainment-oriented Internet use mode did not significantly affect environmental satisfaction. Accordingly, the regression results are consistent with the binary logistic regression analysis results of Model 6. This indicates that the positive effect of the politically-oriented Internet use on public environmental satisfaction is significant and stable. The regression analysis results of entertainment-oriented use are not consistent with the proposed hypothesis that “entertainment-oriented Internet use reduces public environmental satisfaction.” Therefore, H2 is not valid, and the “time substitution hypothesis” is not supported in this study.

The regression results in Table 3 indicate that control variables such as politically-oriented Internet use or entertainment-oriented Internet use, the individual’s education level, urban and rural type, political identity, nationality, unit nature, marital status, per capita nitrogen oxide emissions, industrial solid waste emissions, and grassland area in the regional and provincial levels significantly affected environmental satisfaction. Specifically, in terms of education level, the group that received primary, secondary, and higher education had significantly lower environmental satisfaction than the group that never received education. In terms of urban and rural types, the effect of urban and rural settlements on environmental satisfaction was significantly negative at 1% significance level. This implies that the environmental satisfaction of urban residents was significantly lower than that of rural residents. In terms of political identity, both types of Internet use significantly positively affected the environmental satisfaction of party members. In terms of nationality, politically-oriented and entertainment-oriented Internet use significantly negatively affected environmental satisfaction of Han people. In terms of unit nature, politically-oriented Internet use and entertainment-oriented Internet use significantly positively affected working groups in the public sector. In terms of marital status, Internet use significantly negatively affected environmental satisfaction of married people. In terms of regions, the effect of Internet use on the environmental satisfaction of residents in the central and western regions was significantly lower than that in the eastern regions. In terms of per capita NOx emissions, Internet use was significantly negatively related to environmental satisfaction of the public: environmental satisfaction decreased by 54.932 units for each ton of per capita NOx emission increase. Although the amount of industrial solid waste and grassland area significantly affected environmental satisfaction, the unit change was not obvious.

**Table 3:** Estimated results of the impact of internet use on environmental satisfaction.

Environmental		OLS			Logistic	
satisfaction	m1	m2	m3	m4	m5	m6
Politically-oriented Internet use	0.026***		0.034***	0.033***		0.030***
	(2.93)		(3.15)	(3.46)		(2.63)
Entertainment-oriented Internet use		0.005	-0.013		0.021**	0.005
		(0.50)	(-1.15)		(2.33)	(0.51)
Age	0.005	0.002	0.004	0.001	0.001	0.002
	(1.54)	(0.58)	(1.02)	(0.41)	(0.16)	(0.55)
Gender	0.110	0.120*	0.096	0.056	0.083	0.062
	(1.59)	(1.72)	(1.36)	(0.79)	(1.17)	(0.87)
Primary education (illiterate)	-0.259*	-0.258*	-0.249*	-0.095	-0.106	-0.098
	(-1.72)	(-1.71)	(-1.65)	(-0.75)	(-0.84)	(-0.78)
Secondary education	-0.337**	-0.293*	-0.312**	0.023	0.029	0.013
	(-2.27)	(-1.95)	(-2.08)	(0.18)	(0.23)	(0.10)
Higher Education	-0.299*	-0.186	-0.279	0.353**	0.427**	0.345**
	(-1.71)	(-1.07)	(-1.58)	(2.02)	(2.47)	(1.96)
Income	-0.001	0.004	-0.001	0.004	0.008	0.004
	(-0.10)	(0.36)	(-0.10)	(0.36)	(0.76)	(0.36)
Urban and rural	-0.501***	-0.482***	-0.496***	-0.238***	-0.227***	-0.240***
	(-6.82)	(-6.56)	(-6.74)	(-3.28)	(-3.13)	(-3.30)
Political identity	0.331***	0.372***	0.328***	0.325**	0.361***	0.325**
	(3.19)	(3.61)	(3.16)	(2.53)	(2.84)	(2.54)
Nationality	-0.501***	-0.490***	-0.501***	-0.420***	-0.411***	-0.420***
	(-3.89)	(-3.81)	(-3.89)	(-3.12)	(-3.05)	(-3.12)
Nature of the unit	0.270**	0.313***	0.257**	0.209	0.261*	0.214
	(2.48)	(2.90)	(2.36)	(1.55)	(1.95)	(1.58)
Marital status	-0.255***	-0.247***	-0.255***	-0.236**	-0.231**	-0.236**
	(-2.86)	(-2.77)	(-2.86)	(-2.46)	(-2.40)	(-2.46)
Family population	0.041	0.041	0.041	0.028	0.028	0.028
	(1.59)	(1.59)	(1.57)	(1.10)	(1.12)	(1.11)
Central region (east)	-0.187*	-0.188*	-0.194*	-0.189*	-0.182*	-0.187*
	(-1.78)	(-1.78)	(-1.84)	(-1.77)	(-1.70)	(-1.74)
Western region	-0.493***	-0.498***	-0.502***	-0.412***	-0.403***	-0.408***
	(-3.83)	(-3.87)	(-3.90)	(-3.22)	(-3.15)	(-3.19)
NOx emissions per capita	-54.932***	-54.749***	-54.941***	-35.019**	-34.814**	-35.026**
	(-2.89)	(-2.88)	(-2.89)	(-2.01)	(-2.00)	(-2.01)
Harmless treatment rate of domestic waste	-0.002	-0.000	-0.002	0.026**	0.027**	0.026**
	(-0.13)	(-0.03)	(-0.14)	(1.98)	(2.09)	(1.98)
Total wastewater discharge	0.000	0.000	0.000	-0.000	-0.000	-0.000
	(0.11)	(0.15)	(0.08)	(-0.26)	(-0.20)	(-0.25)
Output of industrial solid waste	0.000**	0.000**	0.000*	0.000	0.000	0.000
	(1.98)	(1.98)	(1.90)	(0.42)	(0.52)	(0.45)
Grassland area	0.000*	0.000*	0.000*	0.000**	0.000**	0.000**
	(1.70)	(1.70)	(1.72)	(2.20)	(2.17)	(2.19)
Constant term	8.050***	8.020***	8.175***	-0.967	-1.146	-1.020
	(5.24)	(5.19)	(5.29)	(-0.73)	(-0.86)	(-0.77)

Note

(1) * * * p < 0.01, * * p < 0.05, * p < 0.1, the brackets are robust standard errors

(2) The variable name in parentheses is a reference group

(3) Models 1, 2, and 3 are multivariate linear regression models, whereas models 4, 5, and 6 are binary logistic regression models.

Notably, the results of the collinearity test revealed that none of the linear regression models had the problem of multi-collinearity (VIF values were less than 4).

### 4.3 Mediating effect analysis

In this study, the mediating effect was further analyzed to verify whether the two types of Internet use affected environmental satisfaction of the public through government trust.

This study uses stepwise regression to assess the mechanism of the mediator. First, whether the independent variable significantly affected the dependent variable statistically was verified. Second, whether the independent variable significantly affected the mediator was assessed. Third, the effect on the dependent variable and the direction of the effect after the mediator and independent variable were included in the regression model were verified. Specifically, the effect of Internet use modes on environmental satisfaction was verified. Thereafter, the effect of politically-oriented Internet use and entertainment-oriented Internet use on government trust was assessed. Finally, government trust was added to the model, with politically-oriented Internet use as the core independent variable and the model with entertainment-oriented use as the core independent variable. If the regression results show a small or significantly reduced estimated coefficient of the independent variable, it can be inferred that Internet use affects environmental satisfaction through the mechanism of government trust.

As presented in Table 4, models 1 and 5 are the OLS estimates of the effect of politically-oriented Internet use and entertainment-oriented Internet use on environmental satisfaction. Models 2 and 6 are the OLS estimates of the effect of politically-oriented Internet use and entertainment-oriented Internet use on government trust. Model 3 is the OLS estimate of the effect of government trust on environmental satisfaction. Models 4 and 7 are OLS estimates of the effect of politically-oriented Internet use and entertainment-oriented Internet use after they are included with government trust. Specifically, Model 1 indicates that politically-oriented Internet use significantly positively affected environmental satisfaction, with an estimated coefficient of 0.026. This implies that for every additional unit of politically-oriented Internet use, environmental satisfaction of the public will increase by 0.026 points. Model 2 indicates that the politically-oriented Internet use significantly positively affected government trust at 1% significance level. This indicates that government trust will increase by 0.018 points for every additional unit of politically-oriented Internet use. Model 3 indicates that government trust significantly positively affected environmental satisfaction at a significance level of 1%. This implies that environmental satisfaction increases by 0.305 points for each unit of government trust. When politically-oriented Internet use and government trust were included in the regression model, the absolute value of the coefficient of Model 4 decreased from 0.026 to 0.023. This indicates that government trust plays an intermediary role in the causal relation between politically-oriented Internet use and public environmental satisfaction. The regression results of Model 5 revealed that entertainment-oriented Internet use did not significantly affect environmental satisfaction of the public. The estimation results of Model 7 indicated no significant effect after adding the mediator variable. This implies that government trust has no mediating effect in the relation between entertainment-oriented Internet use and environmental satisfaction. Thus, H3 is verified, implying that government trust plays an intermediary role in the relation between politically-oriented use and environmental satisfaction. However, H4 is not valid, implying that the intermediary path of entertainment-oriented Internet use to reduce the public’s government trust and thus reduce environmental satisfaction is not established.

**Table 4:** Intermediate effect estimation results.

	Model (1)	Model(2)	Model(3)	Model(4)		Model(5)	Model(6)	Model(7)
	Environmental satisfaction	Government trust	Environmental satisfaction		Environmental satisfaction	Environmental satisfaction	Government trust	Environmental satisfaction
Politically-oriented Internet use	0.026***	0.018***			0.023***			
	(2.93)	(3.38)			(2.68)			
Entertainment-oriented Internet use						0.005	-0.013**	0.012
						(0.50)	(-2.55)	(1.29)
Government trust			0.305***		0.304***			0.306***
			(15.87)		(15.81)			(15.93)
Control variable	Controlled	Controlled	Controlled		Controlled	Controlled	Controlled	Controlled
Constant term	8.050***	12.659***	4.105***		4.101***	8.020***	12.826***	3.972***
	(5.24)	(15.48)	(2.70)		(2.70)	(5.19)	(15.65)	(2.60)
Observations	4,877	9,777	4,877		4,877	4,877	9,777	4,877
R^2^	0.028	0.043	0.085		0.086	0.026	0.042	0.085

## 5. Discussion

This study considered different orientations of Internet use on the basis of CSS2019 and proposed assumptions according to the “doctrine of empowerment” and “time substitution hypothesis” to explore the effect of Internet use on public environmental satisfaction. Furthermore, government trust was introduced to assess whether it is an important factor mediating the relation between Internet use and environmental satisfaction. The findings are discussed in the following paragraphs.

To start, with regard to the descriptive statistical analysis, the results revealed that environmental satisfaction of Chinese public was at a high level. The frequency of politically-oriented Internet use and entertainment-oriented Internet use was relatively high. Furthermore, the results of descriptive statistical analysis revealed that the average score of public government trust in China was 9.45, implying high public trust in the government.

With regard to the benchmark regression results, the first hypothesis about the relationship between orientation of Internet use and public environment satisfaction was partially verified, politically-oriented Internet use significantly improved environmental satisfaction of the public. Specifically, environmental satisfaction of the public can be improved by 0.026 points with a one-point increase in the frequency of politically-oriented Internet use. However, entertainment-oriented Internet use did not significantly affect environmental satisfaction of the public. The study findings support the “doctrine of empowerment” that politically-oriented Internet use can deepen the understanding of public policies and government action, thereby improving public satisfaction. The research results are consistent with the findings of Alfred Tat-Kei Ho [57] and Prior M [58]. The former confirmed that public communication and information are positively associated with citizen’s satisfaction, and the latter asserted that the Internet has widened the gap in political knowledge and voting between the news-hungry and entertainment-hungry public. The findings do not support the time substitution hypothesis, which implies that although recreational activities in the Internet occupy the public’s time on public affairs, they do not affect public environmental satisfaction. Norris proposed to examine not only the duration of media use, but also to distinguish the relationship between the type of media content and public affairs concerns and public affairs participation [59]. Therefore, it makes sense to argue crucial role of Internet use for different purposes in the correlation between analysis variables, breaking the phenomenon that existing studies divide Internet use based on objective quantities (e.g., duration and frequency) and emphasizing the construction of Internet use types by individual subjective preferences (e.g., content of Internet use and purpose of Internet use).

With regard to the mediation effect analysis, the results revealed that government trust played a partial mediating role in the relation between politically-oriented Internet use and environmental satisfaction. This implies that politically-oriented Internet use strengthens the public’s trust in government, thereby improving their environmental satisfaction. However, government trust did not play an intermediary role in the relation between entertainment-oriented Internet use and environmental satisfaction of the public. The second hypotheses were partially supported. The present findings confirm the claims of the “media virtuous circle theory”, which emphasizes positive effects of Internet use on government trust, supporting previous research. Caroline and Karon suggest that the use of new media such as the Internet has a significant effect on enhancing public political trust and raising positive public perceptions of other aspects of local government work [60]. Ceron. A. observed that survey data from 27 European countries indicated a real difference between the messages delivered by online news media and social media. The author confirmed that browse online news is positively correlated with government trust, while social media is associated with lower government trust [61]. Following the “expectation-response” path, this study confirms that government trust positively affects environmental satisfaction, which is consistent with the results of Coulibaly T et al., that is high public trust in the government helps government adopt more environmental protection behaviors that meet the needs and expectations of the public [62]. The present findings provide a complete picture of the interrelation between different orientations of Internet use, government trust, and environmental satisfaction.

Furthermore, in combination with doctrine of empowerment and media virtuous circle theory, gatekeeper theory could provide a more sophisticated understanding of the present findings. According to this theory, Chinese users are more likely to focus on positive information about the government environmental governance practices and environmental conditions from Internet, and have more positive views about the government and the environment. This study further emphasizes that in the network with strong government control, Internet use that actively cares about social news and public affairs has a positive impact on government trust and environmental satisfaction, thereby proving the important role of the Internet in solving environmental problems.

## 6. Implications

Theoretically, the present study extends the literature by examining how orientation of Internet use is associated with public environmental satisfaction. First, the present findings highlight the importance of differentiating these variables, examining how two types of Internet use are linked to public environmental satisfaction offers a more complete picture of the interrelations among these variables. Secondly, this study responds to the controversy between the “doctrine of empowerment” and the “time substitution hypothesis,” as well as the “media inhibition theory” and the “media virtuous circle theory” by examining the correlation between orientations in Internet use and environmental satisfaction by linking the data with the theories, the present study inspires a more comprehensive understanding of orientations in Internet use directly and indirectly influences the public environmental satisfactions. Moreover, this study also demonstrates the applicability of these theories in the Chinese context, which serves to support relevant studies in the future.

Practically, the findings of the present study indicate that policymakers should take the positive effects of internet use and government trust into consideration when developing policies to address public environmental satisfaction. The theoretical and empirical analyses suggest that the enhancement of environmental satisfaction depends not only on government performance, but also on government image and public opinion. Gaining government trust through a good image can enhance individuals’ environmental satisfaction. Further, individuals could understand government policies and behaviors through current political news, which can improve their degree of trust and environmental satisfaction. This article provides several implications for future environmental improvement. First, the government should strengthen ecological construction and environmental protection as well as prioritize environmental protection. Second, promoting “government transparency” and “government response”, disclosing information related to environmental governance to the public, expanding channels for residents to gain a fair understanding of government affairs and communicate with the government [41], promoting the operation of government power under public supervision, building a positive image of the government in online information [63] and enhancing public trust in the government. Third, using AI technology for content gate-keeping and monitoring, what is more important is to be able to effectively channel negative information, use Internet platforms and social media to strengthen communication with the public, openly face criticism and questions, and take the initiative to propose solutions.

## 7. Conclusion

Through descriptive statistical analysis, benchmark multiple linear regression analysis, and mediation effect analysis, this study demonstrated that orientation of internet use could influence public environmental satisfaction directly as well as indirectly through the mediating mechanism of government trust. Moreover, we found that politically-oriented Internet use was more strongly directly related to public environmental satisfaction; that politically-oriented Internet use was more strongly related to government trust. These findings inform future studies as to the importance of distinguishing different types of internet use, they emphasize not only the initiative of individuals in receiving information on the Internet, but also the importance of government building information cyberspace. In the digital era, with the addition of artificial intelligence and digital technology, cyberspace is experiencing rapid development, the Internet may affect people’s values or cognition, practitioners could pay more attention to government-related information construction in cyberspace and effects by helping government improving the government trust and enhancing the public environmental satisfaction.

## 8. Limitations and future studies

We acknowledge that our paper inevitably has some limitations. First, public environmental satisfaction is the result of comprehensive factors. This paper mainly investigates the influence of internet use and government trust on public environmental satisfaction, and other influencing factors have not been investigated. Second, Internet use is divided into two types in this paper, and along with the increase in Internet functionality, Internet use preferences could make more detailed divisions. Analyzing the impact of different types of internet use preferences on public environmental satisfaction will be an important direction for further research in the future. Finally, the generalization of the present findings to other areas should be conservative, since this study only includes samples from China. Due to substantial socioeconomic and cultural differences among different regions, this study should be replicated in other areas to develop a full picture of the relationship between orientation of internet use, government trust, and public environmental satisfaction.
